# The Real Meaning of Fresh Air

**Published:** 1917-01

**Authors:** 


### THE REAL MEANING OF FRESH AIR.

Somehow the veil of mystery always casts a peculiar influence on most persons belonging to modern society. There is a subtle charm and potency in many unexplained manifestations of nature or traditional mysterious prejudices. Something of this sort, though beneficial rather than baneful to mankind, still seems to attach itself to the current impressions about "fresh air." Frederic S. Lee[1] writes:

"In these days, we hear much of 'fresh' air and its merits. We have fresh-air funds, fresh-air schools, and fresh-air babies. All are commendable; but while giving to our funds, opening our schools, and putting our babies out of doors, let us clearly understand what constitutes fresh air. The freshness of so-called 'fresh' air lies, not in more oxygen, less carbon dioxide, less organic matter of respiratory origin, and the hypothetical presence of a hypothetically stimulating ozone, but rather in a low temperature, a low humidity, and motion. So far as fresh air itself is concerned, there seems to be nothing more mysterious about it than this."

What, then, are the specific consequences of a vitiated atmosphere, or, as one may more properly express it in the light of present day knowledge, of objectionably high temperatures and humidity? This is the natural formulation of the inquiry on the part of a medical man; for the interest of the thoughtful physician is perhaps more often centered in the causes and manifestation of disease or malaise than in the explanation of beneficial procedures or remedial agents. Some of the aspects of the modern problems of ventilation and the physiologic action of atmospheric conditions, particularly the work of the New York State Commission on Ventilation,[2] have been discussed in *The Journal*.[3] Hot, humid, still air is harmful in various ways. It affects the circulatory system, the rate of the heart beat being increased in warm, humid air, and decreased in cool, dry air. Eastman and Lee have seen the pulse rate increase by 39—from 67 to 106—as the temperature of the air surrounding the subject rose from 23.3 to 43.3 C. (74 to 110 F.), and the humidity from 58 to 90 per cent. Observations on the response of the vasomotor mechanism indicate that a distinct vascular benefit follows from exposing the body to cool, dry air. The influence of the atmospheric conditions on the respiratory system are varied in character. A moderate degree of heat and humidity seems to be without effect on the rate of respiration; but more extreme conditions cause a quickening of the breathing, and this is probably accompanied by more shallow respirations. We have previously [3] referred to the evidence presented by Miller and Cocks showing that exposure to heat increases swelling, redness and secretion in the nasal mucosa, and these effects are more marked when the humidity of the air is high. Exposure to cold reverses these effects. It is not unlikely that some of the extreme conditions produced render the membranes favorable for the development of infectious micro-organisms. At any rate, there can be little question that the mucous-membrane of the respiratory tract is markedly affected by atmospheric conditions.

Furthermore, the investigations of Lee and Scott[4] at Columbia University have indicated that the distaste for physical labor which is felt on a hot and humid day has a deeper basis than mere inclination—the muscles themselves are actually incapable of performing as much work. These observers state that there is evidently correlation between the decreased muscular power and decreased blood sugar, the muscle fuel. This suggests the existence of a physiologic adaptation such that "when it is physiologically fitting that the animal reduce muscular exertion to a minimum, in order that the output of heat may be as low as possible, as in a hot and humid environment, the supply of fuel will be correspondingly lowered."

It is customary to speak of the body temperature as constant, though diurnal fluctuations are known to exist under conditions which are in every respect normal. Lee[1] has called attention to recent findings of the New York commission with respect to an apparent relationship between the body temperature of man and the temperature of his environment, even under the ordinary conditions of living. Unsuspected, for example, was the fact that during summer months the rectal temperature of its subjects at eight a. m., living in their own homes, was conditioned by the average atmospheric temperature of the preceding night. If the temperature had been warm, the body temperature in the morning was high; if cool, the body temperature was low. The variation was about 0.55 degree C. (1 degree F.) for 20 degrees of atmospheric temperature. In any event, the body temperature was lowered by confinement in an atmosphere of 20 C. (68 F.) and 50 per cent. relative humidity, and raised by confinement at 23.9 C. (75 F.) with the same humidity, or still more by 30 C. (86 F.) with 80 per cent. humidity. The actual average body temperatures found at these stages, respectively, were: 36.7, 36.9, 37.4 C. (98, 98.5, 99.3 F.).

In extreme atmospheric conditions, greater elevations of temperature are known to arise. A stay of about three hours in an atmosphere averaging 40.4 C. (104.7 F.) in temperature and 95 per cent. relative humidity may produce a rise of several degrees in the body temperature of a normal adult man. These suggestive facts raise the question as to the possible control of body temperature in febrile diseases by regulation of the temperature of the surrounding air.—*Journal A. M. A.*

---

[1] Lee, F. S.: Recent Progress in Our Knowledge of the Physiological action of Atmospheric Conditions, read before the American Pediatric Society, Washington, D. C., May 8, 1916; Science, August 11, 1916, p. 183.

[2] For a general presentation of some of the results of the commission, including C. E. A. Winslow, D. D. Kimball, F. S. Lee, J. A. Miller, E. B. Phelps, E. L. Thorndike and G. T. Palmer, see *Am. Jour. Pub. Health,* 1915, v. 85.

[3] Ventilation, editorial, *The Journal A. M. A.*, November 7, 1914, p. 1672; Heat, Humidity and Working Power, January 30, 1915, p. 444; Atmospheric Temperature and Immunity Reactions, May 13, 1916, p. 1553.

[4] Lee, F. S., and Scott, E. L.: *Am. Jour. Physiol.*, 1916, xl, 486.

